# Hyaluronidases and hyaluronan synthases expression is inversely correlated with malignancy in lung/bronchial pre-neoplastic and neoplastic lesions, affecting prognosis

**DOI:** 10.1590/1414-431X20154693

**Published:** 2015-08-28

**Authors:** V.K. de Sá, T.P. Rocha, AL. Moreira, F.A. Soares, T. Takagaki, L. Carvalho, A.G. Nicholson, V.L. Capelozzi

**Affiliations:** 1 Departamento de Patologia, Faculdade de Medicina, Universidade de São Paulo, São Paulo, SP, Brasil; 2 Memorial Sloan-Kettering Cancer Center, New York, NY, USA; 3 Departamento de Anatomia Patológica, A.C. Camargo Cancer Center, São Paulo, SP, Brasil; 4 Divisão de Pneumologia, Instituto do Coração, Faculdade de Medicina, Universidade de São Paulo, São Paulo, SP, Brasil; 5 Universidade de Coimbra, Coimbra, Portugal; 6 Royal Brompton and Harefield Hospitals, NHS Foundation Trust, National Heart and Lung Division, Imperial College, London, UK

**Keywords:** Hyaluronidases and hyaluronan synthases, Lung cancer, Pre-neoplastic lung/bronchial lesions, Immunohistochemistry, Morphometry, Prognosis

## Abstract

We collected a series of 136 lung/bronchial and 56 matched lung parenchyma tissue samples from patients who underwent lung/bronchial biopsies and presented invasive carcinoma after lung surgery. The lung/bronchial samples included basal cell hyperplasia, squamous metaplasia, moderate dysplasia, adenomatous hyperplasia, severe dysplasia, squamous cell carcinoma and adenocarcinoma. Matched lung parenchyma tissue samples included 25 squamous cell carcinomas and 31 adenocarcinomas. Immunohistochemistry was performed to analyze for the distribution of hyaluronidase (Hyal)-1 and −3, and hyaluronan synthases (HAS)-1, −2, and −3. Hyal-1 showed significantly higher expression in basal cell hyperplasia than in moderate dysplasia (P=0.01), atypical adenomatous hyperplasia (P=0.0001), or severe dysplasia (P=0.03). Lower expression of Hyal-3 was found in atypical adenomatous hyperplasia than in basal cell hyperplasia (P=0.01) or moderate dysplasia (P=0.02). HAS-2 was significantly higher in severe dysplasia (P=0.002) and in squamous metaplasia (P=0.04) compared with basal cell hyperplasia. HAS-3 was significantly expressed in basal cell hyperplasia compared with atypical adenomatous hyperplasia (P=0.05) and severe dysplasia (P=0.02). Lower expression of HAS-3 was found in severe dysplasia compared with squamous metaplasia (P=0.01) and moderate dysplasia (P=0.01). Epithelial Hyal-1 and −3 and HAS-1, −2, and −3 expressions were significantly higher in pre-neoplastic lesions than in neoplastic lesions. Comparative Cox multivariate analysis controlled by N stage and histologic tumor type showed that patients with high HAS-3 expression in pre-neoplastic cells obtained by lung/bronchial biopsy presented a significantly higher risk of death (HR=1.19; P=0.04). We concluded that localization of Hyal and HAS in lung/bronchial pre-neoplastic and neoplastic lesions was inversely related to malignancy, which implied that visualizing these factors could be a useful diagnostic procedure for suspected lung cancer. Finalizing this conclusion will require a wider study in a randomized and prospective trial.

## Introduction

Lung cancer remains the leading cause of cancer death worldwide. At the time of diagnosis, lung cancer is usually extensive, and despite improvements in therapy, the overall 5-year survival rate for lung cancer patients remains less than 15% (1). The major reasons for the poor prognosis for lung cancer are the lack of effective screening and early diagnosis procedures, the propensity for early metastasis and the inability of systemic therapies to cure patients with widely metastatic disease (2).

Lung cancer is the result of a multi-step accumulation of genetic and/or epigenetic alterations. Better understanding of the molecular mechanisms by which these alterations affect lung cancer pathogenesis could provide new diagnostic procedures and prognostic factors for detection of early-stage or recurrent disease. In this regard, many have investigated molecular markers in pre-neoplastic and neoplastic lesions to gain insight into tumor recurrence and shortened survival (3). Because degradation of the extracellular matrix is important to invasion, cellular motility, and proliferation, several glycosaminoglycans have been targeted as potentially useful tumor markers. Among these, hyaluronidases (Hyal) and hyaluronan synthases (HAS) have shown to be promising markers. Increased hyaluronidase expression has also been reported in colon (4,5), prostate (6), ovarian (7) endometrium (8), breast (9,10), skin (11), and lung (12–14) cancers. HAS is a group of isozymes that synthesizes hyaluronan (HA), a glycosaminoglycan abundant in connective tissue and also present in several types of epithelia (15,16) where it controls cell migration, differentiation and proliferation (17). Three types of HAS are known: HAS-1, HAS-2, and HAS-3 (18). Therefore, upregulated HAS expression is a likely contributor to HA accumulation in tissues, and promotes tumor growth (4) and metastasis, especially when co-expressed with Hyal (19). Different types of Hyal − Hyal-1, Hyal-2, and Hyal-3 - can degrade the long chains of alternating units of N-acetylglucosamine and glucuronic acid into different-sized molecules. Hyal degrades HA into fragments of 20-25 disaccharides, which are needed to activate the MAP-kinase pathway (20). The major mRNA transcript of Hyal-3 is enzymatically inactive and appears to have only a supportive role in Hyal-1 expression (21).

Expression of epithelial HA, Hyal, and HAS is modulated in premalignant and malignant esophageal, breast, ovarian, and lung tumors (22–24). We hypothesized that Hyal and HAS expressions also change in bronchial metaplasia and dysplasia, and in atypical adenomatous hyperplasia, which are thought to be stages in the development of squamous cell lung carcinoma (25,26) and adenocarcinoma, respectively. To test this hypothesis, we collected samples of metaplastic, dysplastic, and atypical adenomatous hyperplasia lesions and malignant lung tumors and stained them for Hyal and HAS.

## Material and Methods

### Histologic specimens

We retrospectively obtained 136 lung specimens of pre-neoplastic lesions from endoscopic biopsies and lung resections removed from 56 patients who were diagnosed with lung cancer and had undergone diagnostic and/or surgical treatment. Endoscopic biopsies or lung resections were performed under general anesthesia in the Hospital das Clínicas, AC Camargo Cancer Center, University of Coimbra, and Brompton Hospital in London, from 2008 to 2011. For endoscopic biopsies, a flexible bronchoscope (model 1Q10, Olympus Corp., Japan) was used. The bronchoscope was directly used at sites of nodules or masses. Typically, three or more samples were obtained. After this procedure, specimens were fixed in 10% buffered formalin for periods of 4-16 h and embedded in paraffin according to the routine of the laboratory involved. Staining by hematoxylin and eosin, and mucicarmine or PAS with diastase was performed on 5-µm sections in all specimens embedded in paraffin and reviewed by two pathologists (VKS and VLC). Slides resulting from the processing of each specimen were obtained from the pathology files of each institution for review and revision. The 2015 World Health Organization lung tumor classification (27) was used to classify basal cell hyperplasia (n=64), squamous metaplasia (n=13), moderate dysplasia (n=14), severe dysplasia (n=9), and atypical adenomatous hyperplasia (n=36). The invasive carcinomas consisted of 25 squamous cell carcinomas, and 31 adenocarcinomas. For evaluation of staining coverage and intensity, the tissue sections were stained for HAS-1, −2, and −3 and Hyal-1, −2, and −3 as described below. Table 1 summarizes the patient characteristics.

**Table:**
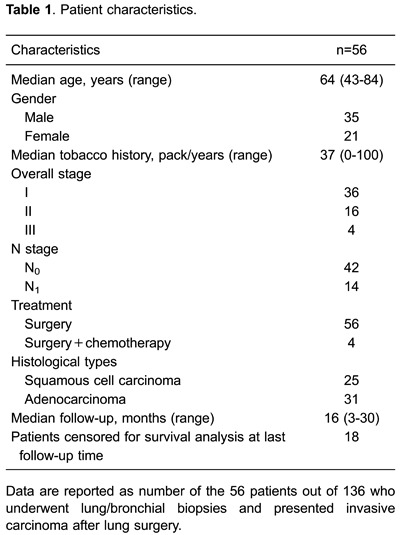

The project was approved by the Ethics Committee for Research Project Analysis (CAPPesq) from the Diretoria Clínica do Hospital das Clínicas and Faculdade de Medicina, Universidade de São Paulo, São Paulo, SP, Brazil.

### HAS and Hyal staining

To avoid antigen decay, serial slide sections from formalin-fixed paraffin-embedded (FFPE) tissues were paraffin coated and stored at 4°C during a median period of 4 years (i.e., from 2008 to 2011) in the different centers included in the study.

Immunohistochemistry was performed to detect Hyal-1, −3 and HAS-1-3 antigens in pre-neoplastic and neoplastic lesions, using monoclonal antibody *Hyal-1* (1D10), Hyal-3 (E-11), HAS-1 (C-14), HAS-2 (S-15), and HAS-3 (E-15), all from Santa Cruz Biotechnology (USA). Briefly, silanized slides containing tissue sections of 3 µm were used in all cases. The sections were deparaffinized in xylol, rehydrated in an alcohol gradient, and stored in 0.05 M sodium phosphate buffer (PBS), pH 7.2-7.4. The sections were then subjected to antigen retrieval in a microwave oven. Next, the slides were incubated overnight with the respective antibodies in concentrations previously established (1:200), washed in 0.05 M PBS, pH 7.2-7.4, and incubated with the secondary antibody, using a large streptavidin-avidin-biotin-peroxidase system (k-0690; Dako A/S, Denmark). Diaminobenzidine (Sigma Diagnostics, USA) was used as a chromogen, and the sections counterstained with hematoxylin. Intense brown cytoplasmic staining in pre-neoplastic and neoplastic lesions was considered positive for Hyal-1 and −3 and HAS-1-3.

### Evaluation of immunostaining

After the immunohistochemical reaction, markers in tumors and pre-neoplastic lesions were quantified using the Automated Cellular Imaging System (ACIS) III instrument (Dako, USA). Briefly, ACIS III consists of an automated digital microscope and a computer with a 26-image capture and image processing system. Each immunohistochemically stained slide was scanned and the images were reviewed on the computer screen. The ACIS III can detect, count and classify cell types based on levels of hue, saturation and brightness. The signal is then converted to number density measurement. The computer software “membranes and cytoplasm”, which is part of the system, was used to analyze protein expression by measuring the staining intensity of the cytoplasm and cell membranes and adjusting the threshold to the pixels showing immunoreactivity or not. The results are reported in continuous variables ranging from 0 to 256. The areas to be analyzed on each slide were selected manually using the selection tools of the ACIS III. For statistical analyses, we used the average of two regions (stroma and tumor) of each case (24).

### Statistical analysis

Statistical analysis was performed using PASW Statistics for Windows, Version 18.0 (SPSS Inc., USA). When necessary, variables were analyzed with the Kolmogorov-Smirnov test to determine the normality pattern. ANOVA tests were used to analyze Hyal-1 and −3 and HAS-1-3 immunoexpression in pre-neoplastic and neoplastic lesions. When non-parametric methods were used, simultaneous comparisons of confidence were corrected with Bonferroni’s posttest. The Spearman test was used to clarify relationships between Hyal-1 and −3 and HAS-1, −2, and −3 staining with pre-neoplastic variables studied. Receiver operation characteristic (ROC) curves were developed to determine optimal cut-off limits that yielded the best possible sensitivity and specificity values. Data on surgical and pathologic parameters, and Hyal and HAS staining inferences, were analyzed by the Cox proportional hazards model, using single-variable analysis (univariate analysis). Stratified Kaplan-Meier analyses were performed on the variables found to be significant in the multivariate Cox proportional hazards model. Results for which P≤0.05 were considered to be significant.

## Results

**Figure 1:**
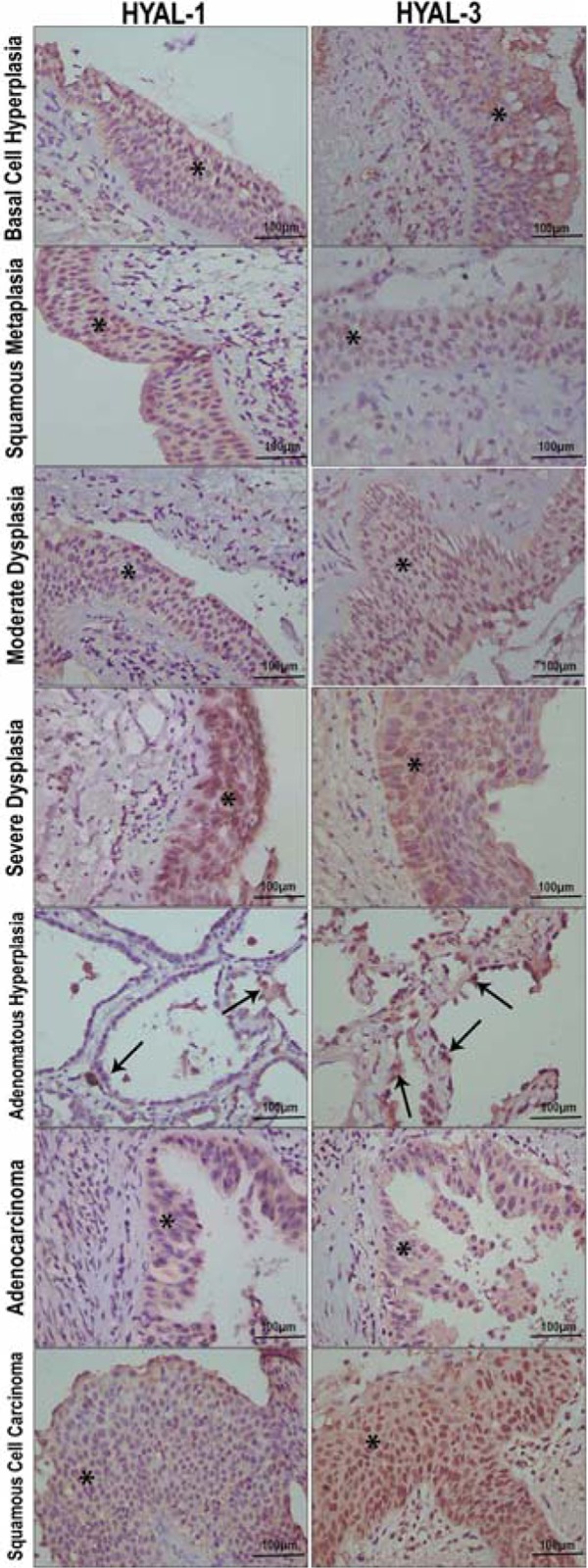
Hyaluronidase (Hyal)-1 and −3 in basal cell hyperplasia, squamous metaplasia, moderate dysplasia, atypical adenomatous hyperplasia, severe dysplasia, squamous cell carcinoma and adenocarcinoma, as shown by immunohistochemistry staining. Hyal-1 was more prominently expressed by epithelial cells in basal cell hyperplasia than in moderate dysplasia, adenomatous hyperplasia, severe dysplasia, squamous cell carcinoma or adenocarcinoma. Hyal-1 expression decreased and became more dispersed as malignancy increased and epithelial cells became less organized. Strong and diffuse expression of Hyal-3 was seen in atypical adenomatous hyperplasia, basal cell hyperplasia and moderate dysplasia. Arrows and asterisks indicate cytoplasmic expression in epithelial cells of pre-neoplastic and neoplastic tissue. Bar: 100 µm.

**Figure 2:**
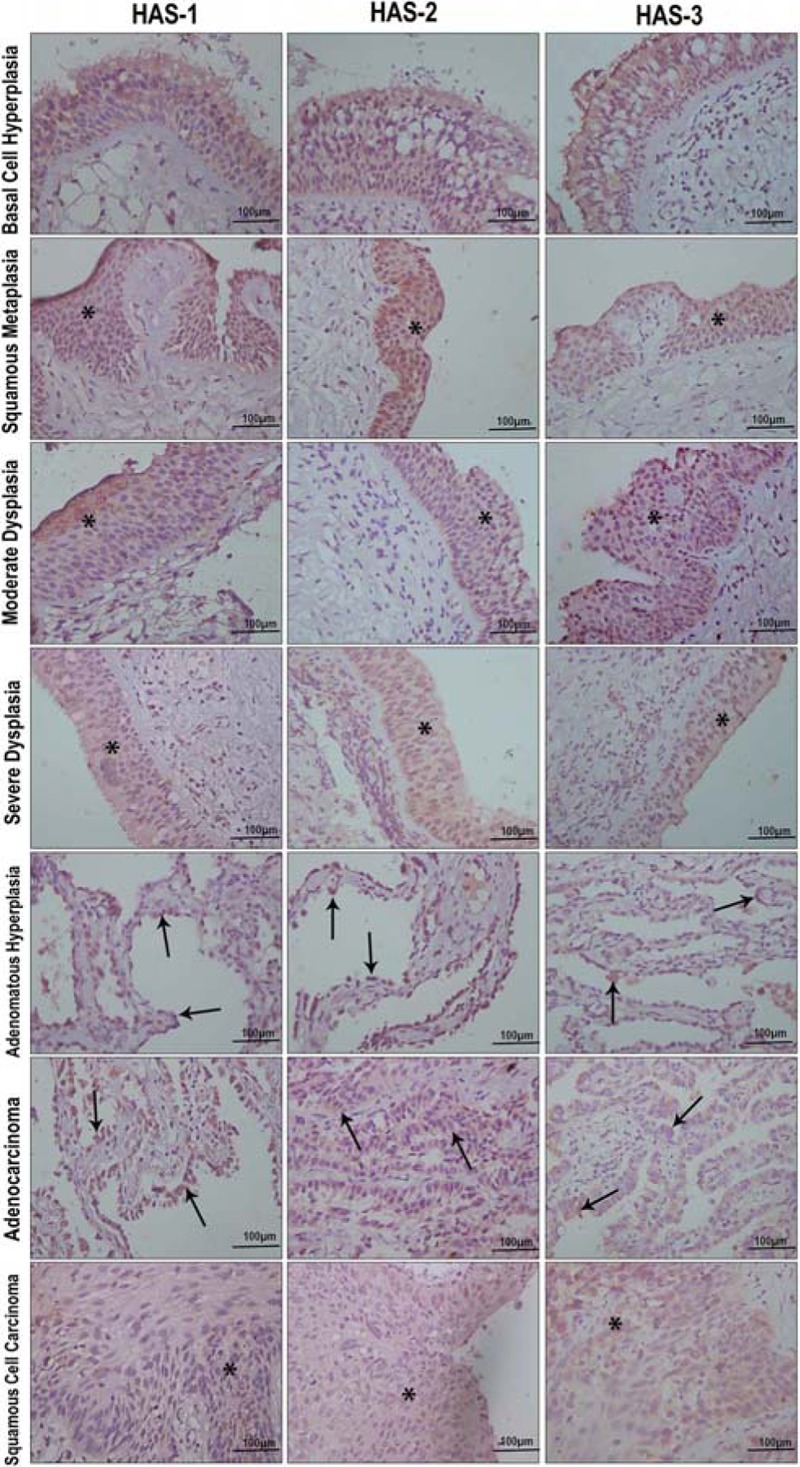
Hyaluronan synthases (HAS)-1, −2, and −3 in basal cell hyperplasia, squamous metaplasia, moderate dysplasia, atypical adenomatous hyperplasia, severe dysplasia, squamous cell carcinoma and adenocarcinoma, shown by immunohistochemistry staining. Similar expression of HAS-1 was seen in basal cell hyperplasia, squamous metaplasia, moderate dysplasia, atypical adenomatous hyperplasia and severe dysplasia. Fewer epithelial cells in squamous cell carcinoma and adenocarcinoma expressed HAS-1. Numerous epithelial cells expressed HAS-2 in severe dysplasia compared to basal cell hyperplasia, squamous metaplasia and basal cell hyperplasia. HAS-3 was prominently expressed in basal cell hyperplasia, atypical adenomatous hyperplasia and severe dysplasia. Few epithelial cells expressed HAS-3 in severe dysplasia compared to squamous metaplasia and moderate dysplasia. Arrows and asterisks indicate cytoplasmic expression in epithelial cells of pre-neoplastic and neoplastic tissue. Bar: 100 µm.

Cells showing Hyal-1- or Hyal-3-positive immunostaining were mainly epithelial cells, whereas most stromal cells showed negative or weak expression (Figure 1). Hyaluronidase-positive staining was localized intracellularly, spreading diffusely throughout the cytoplasm (Figure 2). Immunostaining with specific antibodies for HAS-1-3 showed positive staining in all samples, regardless of the lesion type (Figure 2). The HAS-1-3 proteins were detected homogenously in the cytoplasm and at the plasma membrane (Figure 2).

**Figure 3:**
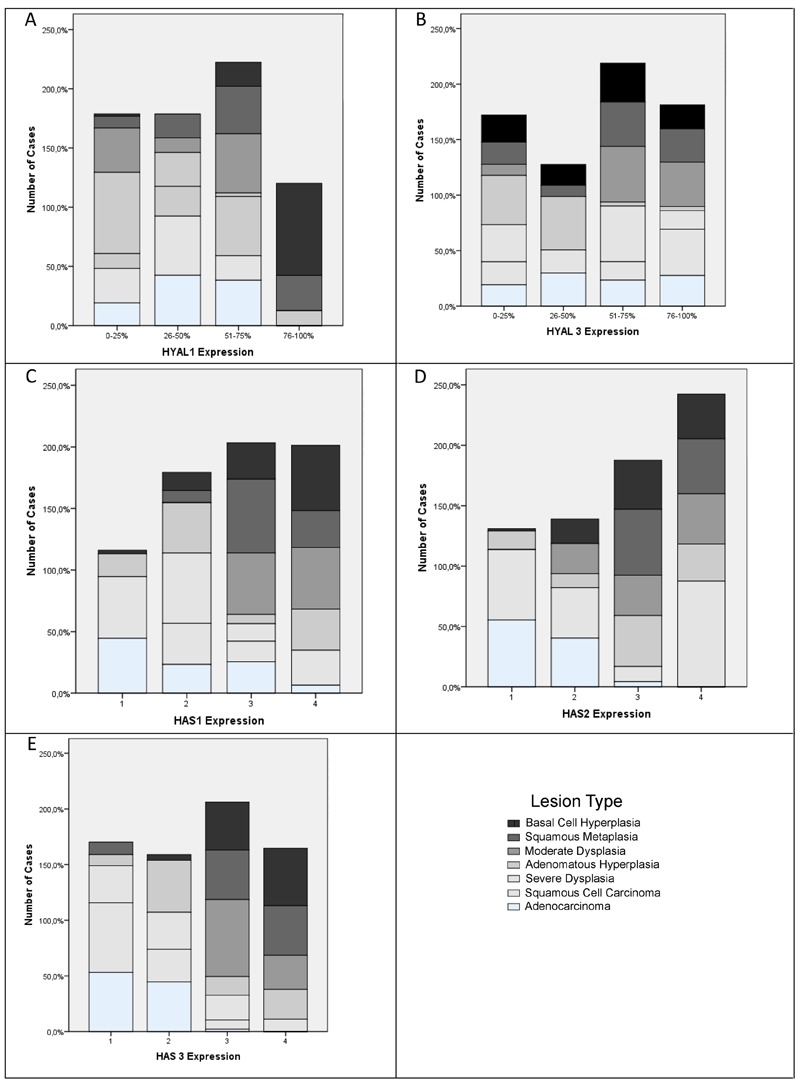
Intensity and coverage of Hyal-1 and −3 and HAS-1, −2, and −3 staining in pre-neoplastic, squamous cell carcinoma and adenocarcinoma. See Results section for complete information about statistical comparisons (ANOVA).

In all tissue groups, Hyal-1-positive epithelial cells ranged from 15.81± 11.16 to 82.56 ± 11.96 (Table 2). The percentage of Hyal-1 was significantly higher in basal cell hyperplasia compared with moderate dysplasia (P=0.02), adenomatous hyperplasia (P=0.0001), severe dysplasia (P=0.05), squamous cell carcinoma (P=0.0001), and adenocarcinoma (P=0.0001; Figure 3A). Adenomatous hyperplasia expressed less Hyal-1 than did squamous metaplasia (P=0.004), squamous cell carcinoma (P<0.01) or adenocarcinoma (P<0.01; Figure 3A). The percentage of Hyal-3-positive epithelial cells ranged from 47.90 ± 22.41% to 79.49 ± 23.51 (Table 2). Hyal-3 staining intensity in epithelial cells was weak in atypical adenomatous hyperplasia, moderate in basal cell hyperplasia and strong in moderate hyperplasia (Figure 3B). The percentage of positive cells in the pre-neoplastic lesions and tumor cells also differed among the HAS isoforms (Table 2; Figures 2 and 3C). The proportion of HAS-1-positive epithelial cells tended to be significantly lower in squamous cell carcinoma and adenocarcinomas than in pre-neoplastic lesions (P=0.05; Figure 3C). HAS-1 percentage in basal cell hyperplasia was 63.14 ± 20.18 whereas average HAS-1-positive cell percentages in other pre-neoplastic lesions were squamous metaplasia: 64.84 ± 16.60; moderate dysplasia: 66.32 ± 13.96; atypical adenomatous hyperplasia: 46.63 ± 31.81; and severe dysplasia: 51.61 ± 22.54 (P<0.05). For squamous cell carcinoma and adenocarcinoma, the proportion of HAS-1-positive cells was typically less than 20 (P=0.0001; Figure 3C). Staining of HAS-2 was significantly higher in severe dysplasia (P=0.002) and squamous metaplasia (P=0.01) than basal cell hyperplasia (Figure 3D). HAS-3 showed significantly greater expression in basal cell hyperplasia than in atypical adenomatous hyperplasia (P<0.05) or severe dysplasia (P<0.05; Figure 3E). Lower HAS-3 expression was found in severe dysplasia than in squamous metaplasia (P<0.01) or moderate dysplasia (P<0.01; Figure 3E).

**Table:**
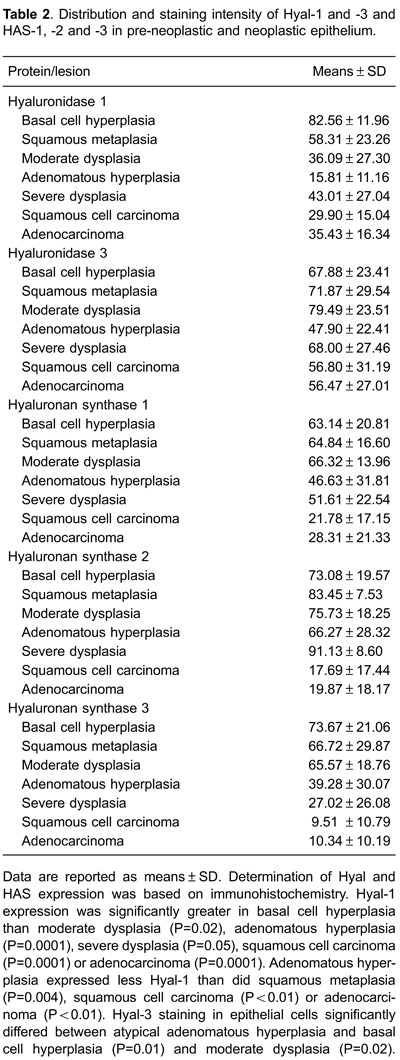

Table 3 shows correlation analyses for Hyal-1 and −3, and HAS-1, −2, and −3 expression and lesion types. A significant direct association was found between Hyal-1 and HAS-1 (R=0.40; P=0.0001), HAS-2 (R=0.40. P=0.0001) and HAS-3 (R=0.50; P=0.0001). Pre-neoplastic and tumor lesions showed inverse associations between Hyal-1 (R=-0.70; P=0.0001), HAS-1 (R=-0.30; P=0.03), HAS-3 (R=-0.45; P=0.0001).

**Table:**
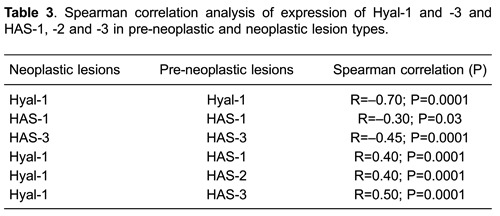

**Figure 4:**
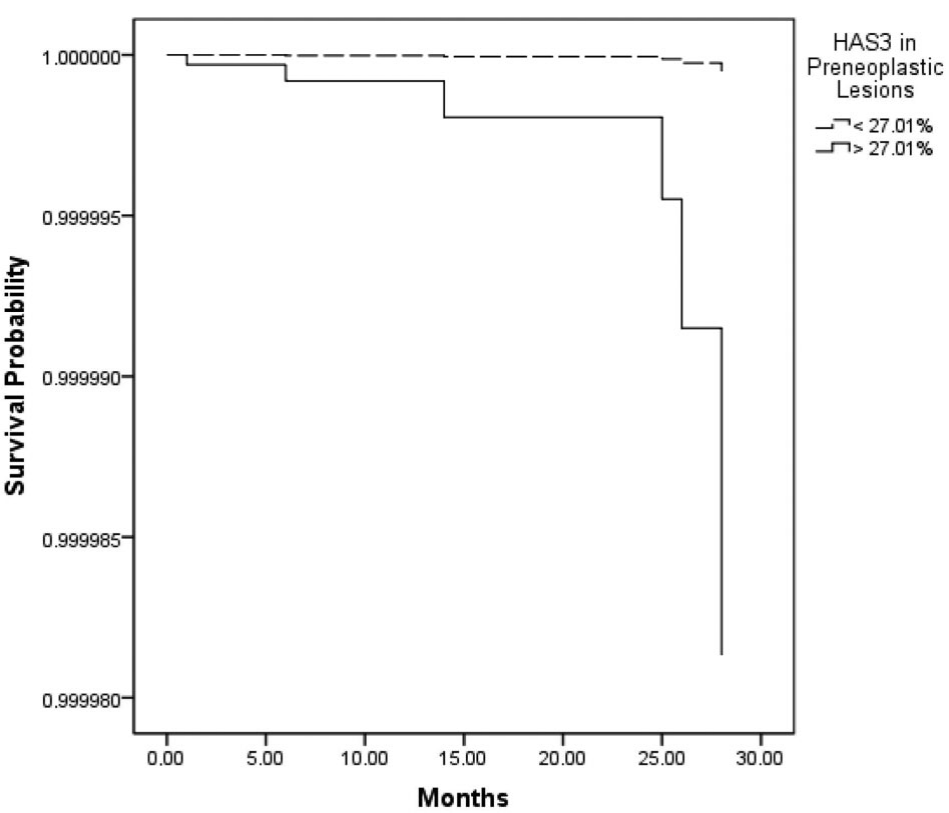
Regression plots of survival probability versus follow-up time in months for all patients. The dashed line indicates the group with <27% HAS-3; the solid line indicates the group with >27% HAS-3.

Comparative Cox multivariate analyses by N stage and tumor histology showed a significant association between poor survival and high pre-neoplastic cell HAS-3 levels (HR=1.19; P=0.04; Table 4). We ranked the cases by ROC curve into two groups with distinctly different average survival times as illustrated by regression plots in Figure 4. The group with <27.01% HAS-3 had a median survival time of 72 months, whereas those with >27.01% HAS-3 had a median survival time of just 52 months after surgery.

**Table:**
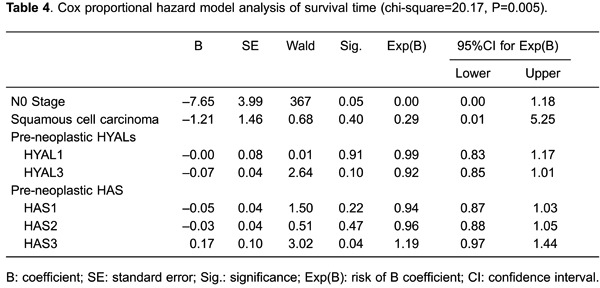

## Discussion

For the present study, we hypothesized that in pre-neoplastic lesions, and squamous cell carcinoma and adenocarcinoma subtypes - i.e., tumors with different behaviors - Hyal and HAS should modulate different malignancy-induced pathways that affect lung cancer carcinogenesis. By IHC staining, we found that Hyal-1 and −3, and HAS-1-3 were significantly overexpressed by epithelial cells in pre-neoplastic lesions compared with tumor epithelial cells. In fact, heterogeneous hyaluronidase expression has been shown in malignancies, and shows promise as an indicator of disease progression.

Antigen decay in archival formalin-fixed paraffin-embedded (FFPE) tissue sections for immunohistochemistry stored at room temperature is a well-known phenomenon which may have affect translational and research studies; length of storage time appears central to this problem (28,29). In the present study, this phenomenon had been previously minimized in the different centers where the samples were obtained for our study, as the serial slide sections from FFPE tissues were paraffin coated and cold stored at 4°C for a median time period of 4 years (i.e., from 2008 to 2011). Additionally, antigens that are nuclear or membranous (e.g., CD3, CD 31, CD117, estrogen and progesterone receptors, Ki67, p53, TTF-1, vimentin) show reduced immunosignals, whereas cytoplasmic antigens (smooth muscle actin, keratins 7, 20, AE1/AE3, 34βE12, Hyal, and HAS) show little antigen decay.

We found that Hyal-1 expression was significantly increased in all pre-neoplastic lesions compared with malignant lesions. Similar results were previously reported by Siiskonen et al. (11), who found the proportion of Hyal-1-positive melanocytic cells was significantly reduced in superficial and deep melanomas and also in lymph node metastases compared with *in situ* melanomas. In our specimens, the staining patterns of Hyal-1 differed between pre-neoplastic and malignant lesions, and the intensity of Hyal-1 in epithelial cells progressively decreased in malignant lesions compared with pre-neoplastic lesions. The presence of hyaluronidase in tumor cells has been shown to increase angiogenesis *in vivo* (12–14). Hyaluronan oligosaccharides produced by hyaluronidases mediate the angiogenic effects (30,31) and may also activate matrix metalloproteinases, thus enhancing tumor invasiveness (32). Interestingly, in a mouse model of prostate cancer, co-expression of a Hyal-1 and a HAS-2 significantly increased angiogenesis (33). Upregulation of Hyal-1 and HAS-1, −2, and −3 in pre-neoplastic lesions was also observed in the present study.

The amount of hyaluronan seems to be biphasic in premalignant and malignant pulmonary lesions. First, in pre-neoplastic lesions, expression of HAS-1-3 in epithelial cells is greater; at this stage, hyaluronan levels vary among benign lesions, such as basal cell hyperplasia and squamous metaplasia. In dysplasia and atypical adenomatous hyperplasia, the proportion of HAS-2-positive epithelial cells is lower than in benign lesions, and at this state the hyaluronan content is also increased in cells of benign lesions. This may indicate accumulation of hyaluronan behind the intact basement membrane before the invasive phase has occurred. Instead, tumor cells show markedly reduced hyaluronan levels, which is associated with increased Hyal-1 expression. In fact, squamous cell carcinoma originates from the stratified epidermis as such. A similar tendency to increase hyaluronan staining in premalignant or early-stage malignant lesions and decreased staining in more advanced tumors has been reported earlier in squamous cell carcinomas of oral (34), laryngeal (35), esophageal (23), and skin (36) epithelium, all originating from stratified epithelium. Although adenocarcinoma originates from alveolar epithelial cells and not from stratified epithelium as such, a similar tendency to increased hyaluronan staining in premalignant or early-stage malignant lesions and decreased staining in more advanced tumors has been reported in adenocarcinomas of oral (34), laryngeal (35), esophageal (22), and skin (11) epithelium, all originating from stratified epithelium.

Our data show, for the first time, the biphasic pattern of hyaluronan metabolism in lung tumors, and reveals increased hyaluronan synthesis in premalignant lesions followed by reduced hyaluronan content in squamous cell carcinoma and adenocarcinoma as a consequence of decreased HAS expression and increased degradative Hyal-1 and −3. Further studies are needed to clarify the prognostic power of Hyal upregulation and HAS downregulation in lung tumors.
